# Mulberry Leaf Flavonoids Improve Milk Production, Antioxidant, and Metabolic Status of Water Buffaloes

**DOI:** 10.3389/fvets.2020.00599

**Published:** 2020-09-04

**Authors:** Mengwei Li, Faiz-ul Hassan, Zhenhua Tang, Lijuan Peng, Xin Liang, Lili Li, Kaiping Peng, Fang Xie, Chengjian Yang

**Affiliations:** ^1^Key Laboratory of Buffalo Genetics, Breeding and Reproduction Technology, Ministry of Agriculture and Guangxi Buffalo Research Institute, Chinese Academy of Agricultural Sciences, Nanning, China; ^2^Institute of Animal and Dairy Sciences, University of Agriculture, Faisalabad, Pakistan

**Keywords:** mulberry leaf flavonoids, milk yield, antioxidant enzymes, metabolic hormones, heat shock proteins, heat stress

## Abstract

This study was aimed to evaluate the effect of mulberry leaf flavonoids (MLF) on oxidative stress, metabolic hormones, and milk production in Murrah buffaloes. Forty multiparous Murrah buffaloes (4 ± 1 lactations) with similar body weight (average 600 ± 50 Kg) and stage of lactation (90 ± 20 d) were randomly selected for this trial. Four treatment groups (10 buffaloes per group) with different doses of MLF included; control (0 g/d), MLF15 (15 g/d), MLF30 (30 g/d), and MLF45 (45 g/d). Buffaloes were fed with total mix ration consisting of grass (*Pennisetum purpureum schum*), brewery's grain and concentrate mixture for 5 weeks. Meteorological data including ambient temperature and relative humidity were recorded using the online dust monitoring system to calculate temperature-humidity index (THI). After 1 week of the adaptation, milk yield was recorded daily while physiological parameters (respiratory rate, rectal, and body surface temperature), and milk composition were measured weekly. At the end of the trial, blood samples were collected to analyze serum metabolic hormones including estradiol (E2), growth hormone (GH), prolactin (PRL), Tri-iodothyronine (T3), and Thyroxine (T4). Moreover, serum heat shock proteins (HSP), antioxidants enzymes including malondialdehyde (MDA), total antioxidant capacity (T-AOC), superoxide dismutase (SOD), catalase (CAT), and glutathione peroxidase (GSH-Px) and blood biochemical indices were also analyzed. Results revealed a decrease (*P* = 0.012) in serum MDA level while increasing (*P* < 0.01) the HSP and serum GHS-Px contents in supplemented buffaloes. Treatment showed a linear and quadratic decrease (*p* = 0.001) in the serum T-AOC while reducing CAT contents linearly (*p* = 0.012) as compared to the control. However, no effect of treatment on serum SOD content was observed. Treatment resulted a linear increase (*p* = 0.001) in serum GH and PRL hormones while increasing serum E2 levels linearly (*P* < 0.001) and quadratically (*P* = 0.025). Treatment increased (*p* = 0.038) the daily milk yield as compared to the control. However, increase (*P* < 0.05) in serum T3 and T4 contents, fat corrected milk (4%) and milk protein (%) was observed only in MLF45. Moreover, we observed no change in serum biochemical indices except insulin which linearly increased (*p* = 0.002) in MLF45. Our findings indicated that MLF at 45 g per day is an appropriate level to enhance milk performance and alleviate heat stress in buffaloes.

## Introduction

Flavonoids belong to a diverse group of plant polyphenols that are widely distributed in different plant species and possess a wide range of biological and pharmacological activities. Most of the plant species used as animal fodders are rich sources of flavonoids along with other polyphenolic contents (1). Due to excellent biological properties, flavonoids are considered efficient feed supplements for livestock to enhance performance (growth and development) and quality of animal products (2). Flavonoids from plant species of genus *Morus* (commonly known as mulberry) which belongs to the family *Moraceae* are famous for their antioxidant potential. The genus *Morus* contains about 68 species and the majority of them are cultivated in Asia (3). China has the largest area of mulberry cultivation (626,000 ha) followed by India (with nearly 280,000 ha). Several other countries [e.g., Thailand and Brazil (ca. 35,000 ha)] still have some mulberry production but on a much smaller scale (4). Owing to its easy propagation and excellent growth characteristics, it can yield leaf biomass of about 25–30 tones/ha/year with a quite less harvesting interval of about 9 to 10 weeks (5). Mulberry leaves contain rich protein content (14–30%) with high *in vivo* dry matter digestibility (75–85%) coupled with a quite luring palatability due to their succulent nature (6). Traditionally, mulberry foliage has been used as alternate forage for livestock in China, mainly due to their rich nutrient profile and flavonoid contents (7). Feeding of mulberry leaves has shown to increase fiber degradation and utilization leading to enhanced milk production in ruminants (8). Moreover, mulberry leaves have shown potent antioxidant and anti-inflammatory properties owing to their rich flavonoid contents (9).

Mulberry leaf flavonoids (MLF) are of great importance due to their excellent antioxidant, biological, and pharmacological activities (10, 11). Major flavonoids in mulberry leaf include isoquercitrin, astragalin, kaempferol, quercetin, and rutin (12). Recent studies have revealed the potential of mulberry-derived flavonoids to effectively improve or sustain animal performance and health (13, 14). The most promising activities of flavonoids in addition to antioxidants; are their potential to modulate different metabolic pathways especially those linked with energy homeostasis in the body. Due to structural homology with estrogenic hormones, flavonoids exhibit similar anabolic functions through modulation of key lipid and carbohydrate metabolic pathways (15, 16). Mulberry leaf extract has shown to upregulate the activities of glycolytic enzymes while downregulating gluconeogenic enzymes through modulation of gene and transcriptional factors involved in glucose homeostasis in the liver of mice (17). Studies have also shown that hydro-ethanolic extract of mulberry leaves significantly reduced the level of lipid peroxidation and adipocyte size in the liver. Moreover, combined mulberry leaf and fruit extract significantly reduced the obesity-related inflammation and oxidative stress in the high-fat diet obesity model (18).

Flavonoids also possess a remarkable ability to enhance non-specific immunity while reducing oxidative stress by promoting the activities of superoxide dismutase (SOD) and glutathione peroxidase (GSH-Px) while lowering the MDA level (19). This activity of flavonoids is mainly attributed to their action as a reducing agent and a hydrogen donor to scavenge reactive oxygen species (ROS) efficiently by removing the peroxide and superoxide radicals (19). Supplementation of MLF significantly improved overall antioxidant capacity while reducing the oxidative stress in *E. coli* challenged pre-weaning calves (20).

Mulberry leaf flavonoids have been extensively studied in mice and monogastric animal models, but studies on ruminants especially dairy animals, are limited. The MLF have also shown to increase the apparent digestibility of organic matter (OM) and neutral detergent fiber (NDF), while reducing methane emission in sheep (7). We hypothesized that owing to their effective antioxidant activity, MLF can effectively reduce the oxidative stress posed by the hot and humid climate of southern China. Moreover, their homology with anabolic steroids can enhance the secretion of metabolic hormones which can subsequently affect lactogenesis (21). The alleviation of oxidative stress coupled with increased secretion of metabolic hormones can synergistically lead to desirable effects on antioxidant status, nutrient metabolism, and milk production performance of buffaloes. Therefore, this study was designed to evaluate the effect of MLF supplementation on antioxidant parameters, metabolic hormones, and milk production of buffaloes under the hot and humid climate of South China.

## Materials and Methods

### Materials

Extract of mulberry (*Morus alba*) leaves (flavonoids 5%) was purchased from Xi'an Feida Biotechnology Co. Ltd., Xi'an, China. The commercial MLF extract mainly consisted of flavones (65.0%), flavonols (20.0%), and other polyphenols (15.0%).

### Geographical Location and Environmental Conditions

**Figure 1:**
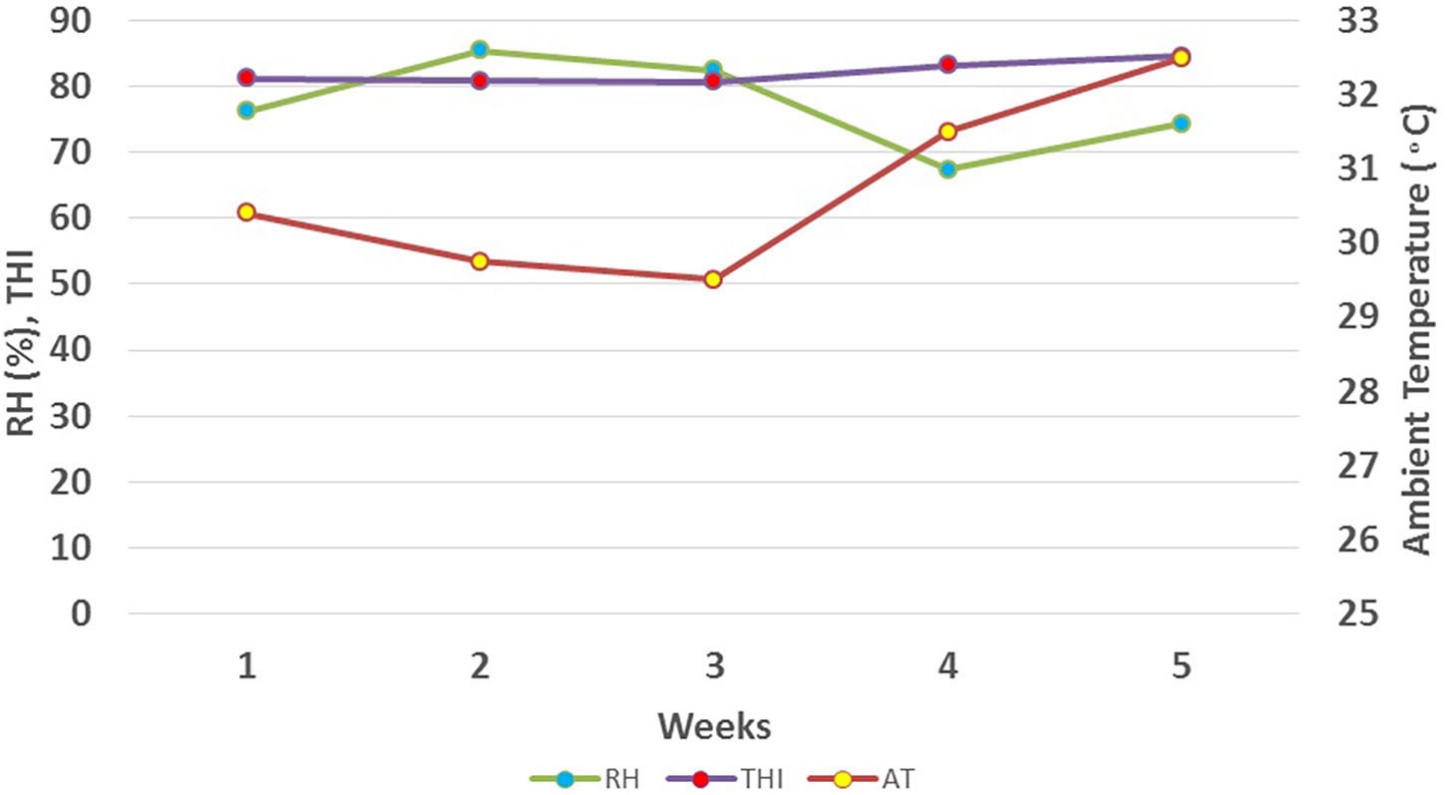
Average weekly ambient temperature, relative humidity, and THI during study period.

This study was conducted from June to July 2019 at the Guangxi Buffalo Research Institute, located in Nanning, South China (N 22° 53′22.59″N, E 108°21′51.19″E; 122 meters above sea level). We used an online dust monitoring system (Shenzhen Greenford Environmental Technology Co., Ltd.) to record weather data in real-time, mainly including air temperature (AT, °C) and relative humidity (RH, %), with an interval of 30 min, at an installation height buffalo's body. Environmental variables were recorded twice daily in the morning (at 8.00 A.M) and afternoon (at 2.30 P.M) during the study period (Figure 1). Average daily temperature and relative humidity were used to calculate Temperature Humidity Index (THI) using the following formula proposed by Thom (22);

$$\begin{array}{l} \text{THI}=\text{AT}+0.36\text{ DPT}+41.5 \end{array}$$

Where AT is the air temperature and DPT is the dew point temperature of the buffalo shed.

### Animals, Diets, and Experimental Design

All procedures used in this experiment were approved by the Ethics committee of the Chinese Academy of Agriculture Sciences, Guangxi Buffalo Research Institute, China. Forty multiparous Murrah buffaloes (4 ± 1 lactations) with an average body weight of 600 ± 50 Kg with the almost same stage of lactation (90 ± 20 days) were randomly selected for this trial. Three levels of MLF were compared in a complete randomized design to evaluate their effect on serum metabolites, stress proteins, antioxidant parameters, and milk production performance of Murrah buffaloes. Four treatment groups (10 buffaloes per group) with different doses of MLF included; MLF15 (15 g/d/head), MLF30 (30 g/d/head), MLF45 (40 g/d/head), and control group (0 g/d/head). Buffaloes were fed with total mix ration (TMR) consisting of grass (*Pennisetum purpureum schum*), brewery's grain and concentrate mixture for 5 weeks. The analysis of crude protein in TMR was performed according to AOAC procedures (23). The TMR samples were also analyzed for neutral detergent fiber (NDF) and acid detergent fiber (ADF) as described previously (24) using an ANKOM2000 Fiber Analyzer Unit (ANKOM Technology Corp., Macedon, NY, USA). Neutral detergent fiber content was analyzed with heat-stable α-amylase and sodium sulfite per sample in the neutral detergent solution. The NDF and ADF were expressed inclusive of residual ash. The gross energy (GE) of rations was determined by a bomb calorimeter (PARR Calorimeter, USA). Details of the chemical composition of the experimental diet are given in Table 1.

**Table 1:** Composition and nutrient levels of basal diets (air-dry basis, %).

**Items**	**Content**
**Ingredients**	
Grass (*Pennisetum purpureum schum)*	32.82
Brewer's grains	48.28
Corn	9.44
Wheat bran	3.98
Soybean meal	3.03
Limestone	0.26
NaCl	0.37
CaHPO_4_	0.32
NaHCO_3_	0.42
Premix^a^	0.18
Total	100
**Nutrient level^b^**	
CP	15.23
NDF	39.57
ADF	27.98
Gross energy (MJ/kg)	16.26
Ash	9.21

a *The premix provided the following per kg of diets:Vitamin E 3,000 IU, Vitamin D 150 000 IU, Vitamin A 500 000 IU, Cu 1.3 g, Fe 4.0 g, Mn 3.0 g, I 80 mg, Zn6.0 g, Co 80 mg, Se 50 mg*.

b *Measured values*.

### Animal Management and Recording of Physiological Parameters

All animals were managed under similar housing and management conditions. Buffaloes were housed in open-sided buffalo shed during milking time, where they were fed once in the morning and afternoon. For exercise, buffaloes were set free in an adjacent open area with a stocking density of 15 m^2^/head. Free access to water was provided to all buffaloes throughout the day. Fans were installed in the buffalo shed above the animal's height to improve airflow. Buffaloes were allowed 30 min swimming time before milking (Once in each morning and afternoon). All animals were fed with a measured quantity of feed twice daily in the morning and evening before milking for *ad libitum* intake. Nutrient requirement of buffalo was calculated according to the technical standards for feeding of water buffalo in China, issued by Hunan Provincial Quality and Technical Supervision Bureau (25). It is based on the bodyweight of animals and the average quantity of milk produced to meet the nutrient requirements of animals. Feed intake of each buffalo was measured individually by collecting and measuring residual feed daily for consecutive 7 days during the last week of the trial.

Weekly average body surface temperature (BST) was recorded on each Tuesday at 8:00 A.M and 2:30 P.M using an animal infrared thermometer from three different body sites (forehead, left chest, and abdomen). At the same time, rectal temperature (RT) was also recorded using a veterinary rectal thermometer (by inserting in the rectum for 15 s) while respiratory rate (RR) was recorded as times/min by observing thoracic movements using a stopwatch and a counter (for 2 min).

### Milk Yield and Composition

Each buffalo was milked twice with milking machines and daily milk yield was recorded for all groups throughout the experimental period. The first week was given as an adaptation period to animals, after that milk yield for morning and evening was recorded daily for each buffalo, while milk samples for determination of milk composition were collected weekly. Milk composition (Milk total solids, protein, fat, and lactose) was analyzed immediately after milking for morning and evening separately using MilkoScanTM F120 (FOSS, Hillerød, Denmark). Fat corrected milk (FCM) at 4% was calculated by using the following equation (26);

$$\begin{array}{l} \text{FCM}\left(4\%\right)=0.4\times \text{Milk yield}+15\times \left(\text{Milk Fat}/100\right)\times \text{Milk yield} \end{array}$$

### Blood Sampling and Haemtological Analysis

Blood samples were collected on the last day of the trial before morning feeding. Blood samples were taken in 10 ml evacuated tubes from the jugular vein to analyze hematological parameters. Two blood samples were collected from each buffalo; first blood was collected in a plain vacutainer tube (10 ml plain vacuum tubes) for obtaining serum, while the second blood sample was collected in a vacutainer tube containing EDTA as the anticoagulant for hematological analysis. Later on, samples were prepared (blood serum) or analyzed (whole blood samples) in the laboratory directly after receiving them. Blood samples in plain tubes were centrifuged at 3,000 rpm for 15 min, and serum was harvested according to standard methods (27). Whole blood samples were used for further analysis of hematological profile including total protein (TP), albumin (ALB), globulins (GLOB), blood urea nitrogen (BUN), and glucose (Glu) using commercial kits according to manufacturer's instructions.

### Determination of Serum Heat Shock Proteins (HSP), Hormones, and Antioxidant Enzymes

Blood samples were put on ice after collection and immediately transferred to the laboratory for separation of serum. Collected serum was stored at −20°C until further analysis. Expression of serum stress proteins (HSP70 and 90) was determined using ELISA Kits. Moreover, metabolic hormones, including Estradiol (E2), Prolactin (PRL), growth hormone (GH), Triiodothyronine (T3), and Thyroxine (T4) were analyzed using ELISA Kits provided by CUSABIO BIOTECH CO., Wuhan, (China) through ELISA assay (ELISA microplate reader) according to manufacturer's instructions. Level of serum antioxidant enzymes including, T-AOC (A015), MDA (A003-1), T-SOD (A001-1), GSH-Px(A005), and CAT (A007-1-1) was determined through spectrophotometer using the Nanjing Built-in Kits (www.njjcbio.com) according to manufacturer's instructions. Average values for each treatment group are expressed as Mean ± S.E. The coefficient of variation (inter and intra-assay) of these kits was in the same range (<10%).

### Statistical Analysis

Data were subjected to the one-way analysis of variance (ANOVA) in SPSS software (SPSS, 2014). Data were analyzed as a complete randomized design using the following statistical model;

$$\begin{array}{l} {\text{Y}}_{\text{ij}}=\text{X}+{\text{T}}_{\text{i}}+{\text{A}}_{\text{j}}+{\text{e}}_{\text{ij}} \end{array}$$

where X is the overall mean; T_i_ is the fixed effect of treatment (i = control, MLF15, MLF30, and MLF45); A_j_ is the random effect of the animal and e_ij_ is the random error term.

Treatment means were compared using Duncan's multiple range test. Moreover, polynomial contrasts (linear and quadratic) were measured to determine the dose-dependent response of MLF supplementation. Significant effects of the treatment were declared at *p* < 0.05.

## Results

### Meteorological Data

Results revealed that the average ambient temperature was 30.73°C, with a range of 27.5–35°C. Relative humidity ranged from 59 to 88 % with an average of 77%. The temperature-humidity index showed a variation from a minimum value of 78 to a maximum value of 87 with, an average value of 82 during 5 weeks of the study period (Figure 1).

### Effect of Treatment on Physiological and Serum Biochemical Parameters

Results revealed no significant effect of supplementation of MLF on physiological parameters, including body surface temperate and respiratory rate (Table 2). However, significantly higher rectal temperature was observed in supplemented groups (MLF15 and 45) as compared to the control group (38.47 to 38.50 vs. 38.31). Our study revealed no effect of treatment on serum biochemical parameters including TP, ALB, GLB, BUN, and glucose in lactating buffaloes (Table 2). However, MLF45 linearly increased (*p* = 0.002) the insulin level as compared to the control.

**Table 2:** Effect of MLF on the physiological, serum biochemical, and antioxidant parameters of buffalo.

**Parameter**	**Control**	**MLF15**	**MLF30**	**MLF45**	**SEM**	***P*****-Value**		
						**Treatment**	**Linear**	**Quadratic**
**Physiological parameters**								
Body surface temperature (°C)	33.75	33.85	33.82	34.15	0.538	0.956	0.642	0.833
Rectal temperature (°C)	38.31^b^	38.50^a^	38.36^ab^	38.47^a^	0.049	0.047	0.142	0.477
Breathing frequency (times/ min)	21.22	20.33	21.15	21.06	0.732	0.812	0.921	0.592
**Serum biochemical parameters**								
Total protein (g/L)	79.98	80.12	77.86	78.08	2.063	0.839	0.956	0.328
Albumin (g/L)	37.12	37.52	35.38	37.3	1.219	0.570	0.340	0.433
Globulin (g/L)	42.86	42.6	42.48	40.78	1.817	0.867	0.560	0.553
Blood urea nitrogen (mmol/L)	12.9	14.64	16.12	16.54	1.127	0.16	0.923	0.037
Glucose (mmol/L)	4.02	3.9	3.89	3.75	0.176	0.905	0.708	0.431
Insulin (ng/ml)	0.78^b^	0.99^b^	1.01^b^	1.26^a^	0.071	0.011	0.002	0.769
**Serum antioxidant parameters**								
SOD (U/mL)	90.92	83.53	87.31	85.83	4.404	0.065	0.576	0.521
CAT (U/mL)	7.47^a^	5.36^b^	5.58^b^	5.29^b^	0.438	0.001	0.012	0.072
T-AOC (U/ml)	3.68^a^	2.07^b^	1.85^b^	2.24^b^	0.186	0.001	0.001	0.001
MDA (nmol/ml)	5.60^a^	3.26^b^	4.15^ab^	1.39^c^	0.467	0.012	*p* < 0.001	0.665
GSH-Px (U/ml)	639.67^a^	740.94^b^	788.71^b^	509.30^c^	26.177	*p* < 0.001	0.019	*p* < 0.001

a, b, c *Values with different superscripts in the same row differ significantly (p < 0.05)*.

### Effect of MLF on Serum Antioxidant Parameters

Supplementation of MLF revealed no effect on serum SOD levels in lactating buffaloes as compared to the control group (Table 2). Treatment showed a linear and quadratic decrease (*p* = 0.001) in the serum T-AOC while reducing CAT contents linearly (*p* = 0.012) as compared to the control. Moreover, a linear decrease (*p* < 0.001) in the serum MDA levels was observed only with lower (41 %) and high (75%) levels of MLF as compared to control. The decrease in MDA levels with a medium level of MLF supplementation was not significant as compared to control. However, serum GSH-Px linearly increased (*p* = 0.019) with the first two levels of MLF (15 and 30 g/d) as compared to the control. But, the higher level of MLF (45 g/d) resulted in a quadratic decrease (*p* < 0.001) in serum GSH-Px content as compared to control and other treatment groups.

### Effects of MLF on Heat Shock Proteins and Serum Metabolic Hormones

The treatment significantly affected the expression of serum heat shock proteins in buffaloes (Table 3). The level of HSP90 linearly increased (*p* = 0.001) with treatment as compared to the control but the higher level of MLF (45 g/d) exhibited a more pronounced effect on HSP90 levels. However, only MLF45 linearly increased (*p* = 0.04) the expression of HSP70 as compared to the control and other treatment groups. Analysis of metabolic hormones showed a linear increase (*p* < 0.001) in serum estradiol (E2) levels with MLF30 and MLF45 as compared to the other treatment groups. However, a linear increase (*p* < 0.001) in the serum prolactin levels was observed in response to MLF treatment. Similarly, growth hormone also showed a linear increase (*p* = 0.001) in response to MLF supplementation. The first two levels of MLF (15 and 30 g/d) exhibited no effect on serum T3 and T4 levels as compared to the control group. However, the higher level of MLF (45 g/d) increased the T3 (*p* = 0.003) and T4 (both linearly and quadratically, *p* < 0.05) levels as compared to other treatment groups. The MLF45 showed a more pronounced effect on T4 levels as compared to T3 (40 vs. 25% increase as compared to the control group).

**Table 3:** Effect of MLF on serum heat shock proteins and metabolic hormones in lactating buffaloes.

**Parameter**	**Control**	**MLF15**	**MLF30**	**MLF45**	**SEM**	***P*****-Value**		
						**Treatment**	**Linear**	**Quadratic**
HSP90 (ng/ml)	9.39^c^	12.21^b^	13.87^b^	19.76^a^	0.828	0.001	*p* < 0.001	0.101
HSP70 (ng/ml)	1.43^b^	1.55^b^	1.52^b^	1.96^a^	0.143	0.000	0.040	0.294
Estradiole (E2) pg/ml	255.71^bc^	223.04^c^	310.80^b^	375.84^a^	17.721	0.002	*p* < 0.001	0.025
Prolactin (μIU/ml)	452.75^c^	568.08^b^	602.40^b^	779.31^a^	36.925	*p* < 0.001	*p* < 0.001	0.429
Growth Hormone (ng/ml)	6.16^b^	8.16^a^	8.20^a^	9.27^a^	0.381	*p* < 0.001	0.001	0.257
T3 (ng/ml)	1.30^b^	1.28^b^	1.18^b^	1.63^a^	0.126	0.003	0.153	0.099
T4 (ng/ml)	35.78^b^	35.29^b^	34.42^b^	50.25^a^	3.382	*p* < 0.001	0.023	0.042

a, b, c *Values with different superscripts in the same row differ significantly (p < 0.05)*.

### Effect of MLF on Milk Yield and Composition

Results revealed a significant effect of treatment on different milk yield and composition parameters except milk lactose (Table 4). The higher level of MLF increased (*p* = 0.038) the daily milk yield of buffaloes as compared to the control group. A comparison amongst different levels of MLF revealed a similar increase in daily milk yield by the first two levels (MLF15 and MLF30) as compared to the control group (*p* < 0.05). However, the higher level of MLF (MLF45) showed a more obvious increase in milk yield as compared to low and medium levels. The MLF45 linearly (*p* = 0.004) and quadratically (*p* = 0.009) increased the FCM (4%) as compared to other treatment groups. The MLF45 also increased (*p* = 0.02) the milk fat (%) as compared to MLF15 but this increase together with a decrease observed in response to MLF15 and MLF30 was not significant as compared to the control. Low and medium levels of MLF showed no effect on milk protein (%) but, the high level (MLF45) increased (*p* = 0.024) the milk protein (%) as compared to the control. Milk total solids (%) decreased (*p* = 0.035) in MLF15 as compared to MLF45 and control groups.

**Table 4:** Effects of MLF on milk yield and composition of buffaloes.

**Parameter**	**Control**	**MLF15**	**MLF30**	**MLF45**	**SEM**	***P*****-Value**		
						**Treatment**	**Linear**	**Quadratic**
Milk yield (Kg/d)	7.11^c^	7.35^b^	7.37^b^	7.67^a^	0.177	0.038	0.064	0.869
Fat corrected milk yield (4%) Kg/d	9.75^bc^	9.09^c^	9.92^b^	10.86^a^	0.235	0.001	0.004	0.009
Milk protein (%)	4.97^b^	4.78^b^	4.84^b^	5.36^a^	0.305	0.024	0.394	0.278
Milk fat (%)	9.15^ab^	8.25^b^	8.98^ab^	9.44^a^	1.232	0.020	0.779	0.596
Total milk solids (%)	20.59^a^	19.29^b^	20.21^ab^	21.34^a^	1.467	0.035	0.642	0.431
Milk lactose (%)	5.26	5.14	5.25	5.19	0.171	0.086	0.899	0.865

a, b, c *Values with different superscripts in the same row differ significantly (p < 0.05)*.

## Discussion

### Physiological and Serum Biochemical Parameters

Flavonoids have shown to increase the metabolic rate by activation of brown adipose tissue, leading to thermogenesis and enhanced energy utilization. Mulberry leaf flavonoids can mediate the transcriptional signaling of different genes related to fat metabolism (28). An increase in rectal temperature in MLF15 and 45 groups indicate the effect of mulberry flavonoids on energy homeostasis in buffaloes, which is in agreement with earlier studies (29). Studies have reported that supplementation of mulberry leaf increased oxygen consumption and activity of obese mice (29). Moreover, flavonoid contents of mulberry leaf activated the brown adipose tissue (which are rich in mitochondria) through the mediation of up-regulated uncoupling protein 1 (UCP1), leading to release dietary energy as body heat (30). This heat increment may increase rectal temperature and body surface temperature in animals (29). This fact might have attributed to increased rectal temperature observed in the present study. Furthermore, activation of adipose tissues can enhance thermogenesis and energy expenditure leading to improved glucose homeostasis and insulin sensitivity (31, 32). Moreover, MLF have shown to trigger the AMPK-PGC-1α signaling pathway to improve glucose metabolism and enhance mitochondrial function to restore ATP homeostasis in skeletal muscles (33). No effect of MLF on respiratory rate and body surface temperature shows the absence of any adverse effects of MLF on the physiology of buffaloes.

No change in serum biochemical parameters was observed in the present study except insulin levels. The non-significant effect of treatment on blood metabolites is in agreement with earlier studies on beef steers fed with ensiled mulberry leaves and sun-dried mulberry pomace (34). These findings reveal that treatment with MLF showed no adverse effects on liver metabolism in buffaloes, as indicated by no change in serum metabolites. It also implies that the level of MLF used in this study is safe for lactating buffaloes as it did not show any adverse effects on downstream metabolic and physiological functions. Moreover, an increase in the serum insulin level in response to MLF45 is in agreement with earlier studies that reported enhanced insulin secretion and sensitivity in response to intra-duodenal supplementation of quercetin (a major flavonoid present in mulberry leaves) in dairy cows (35). Moreover, quercetin has also shown to increase the number of pancreatic islets, which are responsible for insulin secretion (36). In addition, quercetin enhanced the insulin release in isolated islets of Langerhans *in vitro* in a mice model (37). These findings clearly indicate that MLF can positively affect glucose metabolism and could be beneficial in regard to the metabolic adaption of high-producing dairy animals to early lactation. Additionally, optimum insulin levels and proper action are required to effectively alleviate heat stress and minimize oxidative stress damage (38, 39). Overall our findings revealed that MLF supplementation can increase insulin secretion which can subsequently improve the heat tolerance and milk performance of water buffaloes during heat stress.

### Serum Antioxidant Parameters

Under normal environmental conditions (thermoneutral zone), a balance exists in the oxidant-antioxidant system to maintain body homeostasis. However, exposure to higher ambient temperature disrupts this delicate balance leading to oxidative stress. Heat stress exposes animals to an excessive load of ROS leading to severe oxidative stress that subsequently increases lipid peroxidation and reduces immune response (40, 41). Therefore, it is crucial to maintain an oxidant-antioxidant balance to avoid the adverse effects of oxidative stress. In dairy animals, especially in lactating buffaloes, the effect of this oxidative stress is more pronounced as milk production is also a metabolic activity that generates heat. The MDA is an end product of lipid peroxidation which is commonly used as a biomarker to indicate the degree of oxidative stress and level of free radicals (42). Under oxidative stress conditions, the defense system of a body fails to scavenge a large number of free radicals in time due to reduced activities of antioxidant enzymes (CAT, SOD, GSH-Px) under chronic heat stress (43). Therefore, levels of MDA and antioxidant enzymes in ruminants are commonly used as physiological indicators to determine the degree of oxidative and heat stress (44).

In the present study, the THI value exceeded 80 during the study period which resulted in heat stress in lactating buffaloes as revealed by higher MDA levels in the control group. However, MLF significantly reduced MDA levels in treated buffaloes revealing its potent ability to alleviate oxidative stress. Remarkable decrease in MDA levels up to 75% as compared to the control indicated the useful potential of MLF to alleviate heat-induced oxidative stress. These findings are is in agreement with earlier studies reporting inhibition of ROS by mulberry flavonoids in a dose-dependent fashion (45). Even the mulberry leaf powder or their extracts have shown antioxidant, anti-inflammatory, hypolipidemic, and neuroprotective properties (9, 46, 47). A substantial decrease in MDA levels in treated groups revealed that MLF possess practical potential to decrease lipid peroxidation, as reported previously in mice model (18, 48).

The treatment showed no effect on serum SOD content in the present study. However, it significantly decreased the levels of serum T-AOC and CAT as compared to the control. Moreover, a higher GSH-Px level in MLF15 and MLF30 but lower level in MLF45 was observed as compared to the control. Studies have reported that oxidative stress induces the expression and activity of GSH-Px, hence the higher level of GSH-Px indicates more severe heat-induced oxidative stress (49). A lower level of GSH-Px observed in buffaloes supplemented with a higher dose of MLF (45 g/d) reveals the optimum level of flavonoids required to adequately scavenge free ROS ultimately leading to reduced oxidative stress and subsequent decreased activity of GSH-Px. The decrease in the activity of serum T-AOC and CAT in buffaloes supplemented with MLF revealed a relatively lower level of oxidative stress. This is in agreement with earlier studies on pre-weaning calves supplemented with MLF (20). Moreover, treatment with flavonoids has shown to enhance antioxidant capacity, improve non-specific immunity, and alleviate oxidative stress by increasing SOD and GSH-Px activity while decreasing the MDA concentration (19). It is mainly attributed to the ability of flavonoids to act as reducing agents and hydrogen donors to neutralize ROS and remove hydrogen peroxide and superoxide ions (19). The findings of the present experiment support the earlier studies about the dual functionality of MLF to alleviate oxidative stress; (1) by directly interacting with ROS (2) by increasing the activity of antioxidant enzymes.

### Heat Shock Proteins and Metabolic Hormones

Heat shock proteins are well-known for their ability to mediate heat stress and considered as cellular thermometers. Especially the 70-kDa and 90-kDa heat shock proteins (HSP70 and 90) play a key role in thermo-tolerance. These proteins function as molecular chaperons and have significant roles in cellular thermotolerance, apoptosis, immune-modulation, and heat stress (50). Under oxidative stress conditions, the cellular expression of HSP is up-regulated to minimize the accumulation of denatured or abnormal proteins. The HSP are ubiquitously essential to prevent cell damage and enhance the ability to withstand oxidative and thermal stress (51).

In the present study, MLF significantly increased the serum HSP90 content as compared to the control group. However, a higher HSP90 level was observed in MLF45 while the other two treatment doses showed the same levels. Moreover, lower and medium levels of MLF (15 and 30 g/d) showed no effect on serum HSP70 content but a high level of MLF (40 g/d) significantly increased HSP70 content as compared to the control. These findings revealed a variable effective dose of MLF required to enhance the expression of HSP in buffaloes under heat stress conditions. Earlier studies in mice model have reported that quercetin can effectively upregulate the expression of the HSP (especially HSP70) through mediating the ERK/PPARγ signaling pathways (52). Moreover, the extent of such effects induced by flavonoids on HSP was dependent on the molecular weight (family) of HSP (52). This may be attributed to the fact that we observed slightly variable effects of MLF on the expression of serum HSP70 and 90.

Dietary flavonoids not only act as potent antioxidants but also regulate various signaling pathways to resist heat-induced oxidative stress at the cellular level (53). Moreover, they also possess the ability to enhance the absorption and utilization of dietary nutrients, immune response, and the development of mammary glands as well as lactation performance in animals (54, 55). In the present study, supplementation of MLF enhanced the concentration of serum metabolic hormones including E2, GH, and PRL. This may be attributed to the fact that the molecular structure of flavonoids resembles anabolic steroid hormones (phytoestrogens), which enables them to regulate the secretion of the different endocrine hormones by mediating hypothalamus-pituitary-axis (21). Owing to structural similarities of flavonoids with natural estrogen hormone along with other steroid hormones and their antagonists (56), they possess a great affinity for endoplasm reticulum and subsequent ability to mediate gene expression like estrogens, albeit at a lower affinity (57).

In the present study, a higher dose of MLF (45 g/day) showed more pronounced effects on the secretion of E2, GH, and PRL hormones as compared to low and medium levels of supplementation. We observed an increase of 46, 72, and 50 % in serum concentration of E2, GH, and PRL, respectively, in MLF45 as compared to the control group. The lower and medium levels of MLF (MLF15 and MLF30) had no effect on serum T3 and T4 contents. However, a substantial increase in serum T3 (25%) and T4 (40%) contents was observed with a higher dose of MLF (45 g/d) in buffaloes. It reveals that the effect of MLF on endocrine physiology is dose-dependent which is in agreement with earlier studies (45, 58).

### Milk Yield and Composition

Due to rich nutrient profile, flavonoid contents, and abundant availability, mulberry leaves have been considered as alternate forage for ruminants especially in China (7). Studies have shown that ensiled mulberry leaves and sun-dried mulberry pomace are also potential feed ingredients for animals (59, 60). Mulberry leaf flavonoids have shown to improve the feed intake and growth performance in ruminants. However, no study has explored the effect of MLF on milk yield and composition in dairy cattle and buffalo. Therefore, we will compare our findings with studies supplementing flavonoids from different sources. Our findings revealed a significant increase in the daily milk yield of buffaloes in response to all levels of MLF supplementation as compared to the control group. However, this increase was more pronounced in MLF45 as the other two groups did not show a significant difference between each other. Similarly, FCM (4%) and milk protein (%) was also significantly higher in MLF45 as compared to other treatment and control groups. Similar findings have been reported earlier regarding the increase in daily milk and protein yield and energy corrected milk in response to supplementation of flavonoid-rich diet containing grape seed or grape marc meal extract or green tea or Curcuma in dairy cattle (61, 62).

In the present study, the treatment showed a significant increase in milk fat and protein (%) only with a higher dose of MLF (45 g/d) as compared to the control. Increased milk protein content has been observed in an earlier study in dairy cows supplemented with quercetin (35). In addition, our findings were consistent with higher propionate and total VFA contents observed in earlier studies involving supplementation of MLF in Holstein calves (63). These desirable changes in rumen fermentation may be attributed to the favorable effect of MLF on major cellulolytic bacteria (like *Ruminococcus albus)* as supplementation of ensiled mulberry leaves in the diet of fattening steers showed a significant increase in *R. albus* (60). Such changes in gut bacteria can potentially increase cellulose degradation leading to better rumen kinetics and higher VFA yield. Moreover, diet containing ensiled mulberry leaves and mulberry fruit pomace has shown to increase the relative abundance of amylolytic bacteria (particularly *S. bovis*, and *Ruminobacter amylophilus*) which can positively influence starch degradation in the upper gut and consequently increase the glucose content of the intestine (60). It has also been suggested that mulberry leave and fruit pomace can produce more fermentable glucose in the gut and also positively influence protein utilization by microorganisms subsequently leading to better energetic efficiency in ruminants (59, 60). Moreover, flavonoids (like quercetin) have shown to decrease total protozoa and methanogens to significantly decrease *in vitro* methane production without adversely affecting rumen microbial fermentation (64). These findings collectively suggest that MLF can potentially modulate rumen microbiome to mediate fermentation kinetics subsequently leading to better nutrient utilization and performance in ruminants. It is quite pertinent to mention that in the present study, MLF substantially decreased the abundance of *Prevotella* in the rumen of buffalo (data not presented) in MLF45 as compared to MLF15 and the control. It is well-established that *Prevotella* has a negative correlation with DMI and milk fat content (65, 66). Therefore, a reduction in the abundance of *Prevotella* in MLF45 was well-associated with higher milk fat (%). Moreover, Prevotella species are more specialized for protein degradation, peptide fermentation, and their uptake in the rumen (67). Its reduction in the rumen may have increased the amount of rumen bypass protein leading to enhanced duodenal protein supply that subsequently affected milk protein contents. These findings are in agreement with earlier study reporting reduction of dietary protein degradation under *in vitro* conditions when ryegrass grass was supplemented with rutin (68). In addition, studies have reported that lower *Prevotella* abundance has been observed in high producing cows as compared to low producing dairy cows (69). It is the most likely reason for higher milk yield and protein (%) observed in MLF45 as compared to other treatment groups.

The present study revealed that MLF at 45 g per day is an appropriate dose for supplementation in buffaloes to enhance milk production performance. The dose-dependent effect of MLF observed in the present study is also consistent with earlier studies reporting the higher average daily gain in calves fed with a high level of MLF as compared to lower levels (63, 70, 71). These studies suggested that alleviation of oxidative stress by MLF can impart beneficial effects on animal health and performance as observed on the average daily gain in calves previously. The desirable effects of MLF on lactation performance observed in the present study were well-associated with levels of serum GH and PRL. The galactopoietic effects of GH are well-established as its level is positively associated with milk yield in dairy cattle (72–74). The galactopoietic effects of growth hormone are mediated via increased secretion of GH-releasing factor and insulin-like growth factor-I (IGF-I) in dairy cows (74, 75). Studies have suggested that GH stimulates milk production by partitioning nutrients from adipose tissue and muscle, increasing blood flow to the mammary gland, increasing feed intake, and reducing whole-body amino acid oxidation and urinary nitrogen loss (76). Many studies have shown that the positive effects of GH on milk yield in dairy animals are related to an increase in the proliferation and activity of mammary epithelial cells (77, 78). It may be mediated either through the direct effect of GH on the mammary gland or an indirect effect via increased secretion of IGF-1 (79, 80). Moreover, GH also modulates the ribosomal protein S6 phosphorylation leading to enhanced protein synthesis in the mammary gland ultimately increasing milk protein yield (81, 82). On the other hand, PRL also plays an important regulatory function in mammary gland development, milk secretion, and expression of milk proteins (83–85) and have shown galactopoietic effects in dairy ruminants (86). Moreover, increasing the PRL secretion by injecting a dopamine antagonist (domperidone) was found to increase milk production (87). In addition, more apoptosis was found in the mammary glands of cows when PRL secretion was inhibited (88), suggesting that PRL is a survival factor for mammary cells and important mediator for lactation persistency. Keeping in view of the above-mentioned facts, it is suggested that a more pronounced effect of a higher dose of MLF on milk yield and milk protein (%) observed in the present study is partly attributed to the higher level of GH and PRL found in this group. In addition, higher insulin levels observed in MLF45 were well-associated with higher milk yield, FCM, and milk protein in the present study. Our findings are in agreement with earlier studies as supplementation of quercetin has shown to increase milk protein (%) in dairy cows that was believed to be associated with plasma insulin concentration via the GH-IGF axis (35, 89).

Mulberry leaf flavonoids have shown to increase the nutrient digestibility, dietary metabolizable energy, and rumen fermentation in pre and post-weaning calves (70, 90). The desirable effects of MLF on lactation performance observed in the present study may be attributed to the increased secretion of metabolic hormones and modulation of the insulin/IGF-1 signaling pathway (91). Moreover, flavonoids also possess excellent antioxidant activity, which leads to the restoration of physiological homeostasis at a cellular level like maintaining insulin levels. An increase in insulin secretion and subsequent acceleration in oxidative degradation of sugars eventually leads to enhanced feed intake and positive energy balance under heat stress conditions (92). Moreover, MLF possess functional characteristics like modulation of expression and activities of several enzymes involved in lipid and carbohydrate metabolism, which can ultimately affect animal performance to a greater extent (15, 16). Many studies have also provided the convincing evidence that flavonoids possess the therapeutic potential to improve metabolic parameters possibly through modulating peroxisome proliferator-activated receptor (PPAR)-γ, which plays a crucial role in the regulation of fatty acid and glucose metabolism (93, 94). Furthermore, mulberry leaves have also shown to maintain optimum conditions for rumen fermentation to enhance nutrient digestion and utilization in beef cattle (92). Dietary supplementation of MLF has also been reported to enhance the digestibility of organic matter and NDF in sheep (7). So, all these desirable effects can synergistically contribute to the overall beneficial outcomes in terms of better lactation performance in dairy animals as observed in this study.

According to the best of our knowledge, it is the first study to reveal the excellent antioxidant potential of MLF to alleviate heat stress in lactating buffaloes managed under the tropical environment. The increase observed in daily milk yield, FCM, and milk protein content in response to MLF45 is economically important and indicates the practical feasibility of MLF supplementation in dairy production systems particularly under heat stress conditions. The outcome of the present study suggested that alleviation of oxidative stress significantly contributes to the beneficial effects on metabolic status and lactation performance under a hot and humid climate.

## Conclusions

Dietary supplementation of MLF significantly decreased the oxidative stress marker (MDA) while increasing the serum heat shock proteins, GHS-Px, and insulin contents. However, the treatment decreased T-AOC and CAT while no effect on serum SOD contents was observed owing to reduced oxidative stress in buffaloes. Our study revealed the dose-dependent effect of MLF on serum metabolic hormones (E2, PRL, GH, T3, and T4), daily milk yield, FCM (4%), and milk protein (%) in Murrah buffaloes. The present study concluded that MLF at 45 g per day is the most appropriate dose for supplementation in lactating buffaloes to enhance lactation performance and alleviation of heat-induced oxidative stress during the summer season. However, studies on a larger cohort are required to provide mechanistic insights on the role of MLF on alleviation of oxidative stress, modulation of rumen microbiome and hypothalamus-pituitary axis to mediate metabolic functions and performance in lactating buffaloes.

## Data Availability Statement

The original contributions presented in the study are included in the article/supplementary material, further inquiries can be directed to the corresponding author/s.

## Ethics Statement

The animal study was reviewed and approved by Ethics committee of the Chinese Academy of Agriculture Sciences, Guangxi Buffalo Research Institute, China.

## Author Contributions

CY: conceptualization, funding acquisition, supervision, and validation. ML and FH: data curation. FH and ZT: formal analysis. ML: investigation. ML, LP, KP, LL, and XL: methodology. XL and CY: project administration. ZT, FX, and CY: resources. FH: writing—original draft. FH, ML, and CY: writing—review & editing. All authors contributed to the article and approved the submitted version.

## Conflict of Interest

The authors declare that the research was conducted in the absence of any commercial or financial relationships that could be construed as a potential conflict of interest.
